# Glycemia and the cardioprotective effects of insulin pre-conditioning in the isolated rat heart

**DOI:** 10.1186/s12933-017-0527-5

**Published:** 2017-04-04

**Authors:** Yosuke Nakadate, Hiroaki Sato, Takeshi Oguchi, Tamaki Sato, Akiko Kawakami, Tadahiko Ishiyama, Takashi Matsukawa, Thomas Schricker

**Affiliations:** 1 Department of Anesthesia, McGill University Health Centre Glen Site, Royal Victoria Hospital, 1001 Blvd, Decarie, Montreal, QC H4A 3J1 Canada; 2 Department of Anesthesiology, University of Yamanashi, 1110 Shimokato, Chuo-city, Yamanashi 409-3898 Japan; 3 Operating Theater, Yamanashi University Hospital, 1110 Shimokato, Chuo-city, Yamanashi 409-3898 Japan

**Keywords:** Acute hyperglycemia, Insulin-induced cardioprotection, Stunned myocardium, Isolated rat heart, Cardiac contractility, Phospho-protein kinase B, Tumor necrosis factor-α

## Abstract

**Background:**

While acute hyperglycemia has been shown to mitigate the beneficial effects of ischemic preconditioning, its effect on insulin-induced preconditioning remains unclear.

**Methods:**

The study was designed to test the hypothesis that acute hyperglycemia diminishes the cardioprotective effects following a 20-min pre-ischemic pre-conditioning with insulin in the isolated rat heart using the Langendorff system. Forty hearts were assigned to receive modified Krebs–Henseleit (KH) buffer containing 0.5 U/L insulin and 100 mg/dL glucose (InsG100, n = 10), KH buffer with 100 mg/dL glucose (G100, n = 10), KH buffer supplemented with 0.5 U/L insulin and 600 mg/dL glucose (InsG600, n = 10), or with 600 mg/dL glucose (G600, n = 10). To match the osmotic pressure of the InsG600 group, 27.5 mmol/L of mannitol was added to KH solution in the InsG100 and G100 group. The four groups were perfused with each solution for 20 min prior to 15 min of no-flow ischemia, and during 20 min of reperfusion. Only during the ischemic period the heart was paced at 222 beats/min. Measurements of heart rate, coronary flow and maximum of LV derivative of pressure development (dP/dt max) were recorded. Myocardial phospho-protein kinase B (p-Akt) and tumor necrosis factor-α (TNF-α) levels were assayed by enzyme-linked immunosorbent assay and sandwich ELISA, respectively following reperfusion.

**Results:**

After reperfusion, LV dP/dt max and heart rate in the InsG100 group was significantly higher than that in the other three groups. The myocardial p-Akt level in the InsG100 group was significantly elevated when compared to the InsG600 group at the end of reperfusion. The p-Akt levels in the InsG600 and InsG100 group were significantly higher than in the corresponding non-insulin groups.

**Conclusions:**

Acute hyperglycemia diminishes the cardioprotective effects of insulin preconditioning in the isolated rat heart, possibly mediated through the suppression of myocardial Akt phosphorylation.

## Background

Ischemic and pharmacological pre-conditioning techniques [1] reverse some of the adverse consequences of cardiopulmonary bypass (CPB), i.e. hypothermia, hyperoxia, platelet dysfunction, enhanced neuroendocrine outflow, hyperglycemia and myocardial depression [2]. The mechanisms responsible for these beneficial effects in cardiac surgery involve specific modifications of cell surface receptors, mitochondrial components, and signaling kinases [1]. We recently demonstrated that insulin pre-conditioning improves cardiac contractility and that this cardioprotective influence is associated with the activation of the myocardial phosphatidylinositol 3-kinase/protein kinase B (PI3K/Akt) signaling system [1, 3].

Perioperative hyperglycemia, a typical metabolic feature of the surgical stress response and established risk factor for adverse outcomes after cardiac surgery [4], has long been recognized to inhibit certain signal transduction pathways relevant for cardioprotection [5], to stimulate the production of pro-inflammatory cytokines [6], and to favour coronary vasoconstriction and thrombosis [7]. It remains still unclear, however, whether hyperglycemia also annihilates the benefits of insulin pre-conditioning.

The present study was designed to test the hypothesis that acute hyperglycemia diminishes the cardioprotective effects following a 20-min pre-ischemic pre-conditioning with insulin in the isolated rat heart. We also investigated the role of myocardial phospho-protein kinase B (p-Akt) and tumor necrosis factor-α (TNF-α) as potential mediators.

## Methods

### Langendorff perfusion system

Male Wistar rats (weighing 300–320 g) were anesthetized by intraperitoneal injection of pentobarbital sodium (60 mg/kg body weight). Hearts were excised and promptly immersed in cold modified Krebs–Henseleit (KH) buffer at 4 °C. The aorta was cannulated, and retrograde arterial perfusion was started at a constant pressure of 55 mmHg. The perfusion solution was modified KH buffer containing NaCl (118 mmol/L), NaHCO_3_ (25 mmol/L), KCl (4.7 mmol/L), KH_2_PO_4_ (1.2 mmol/L), MgSO_4_ (1.2 mmol/L), CaCl_2_ (2.0 mmol/L), di-NaEDTA (0.5 mmol/L), and glucose 5.5 mmol/L (100 mg/dL). The KH solution was maintained at 37 °C and gassed with 95% O_2_ and 5% CO_2_. A thin latex balloon was inserted into the left ventricle through the mitral valve and connected to a pressure transducer (TruWave, Edwards Lifesciences, CA, USA) for continuous monitoring of left ventricular (LV) pressure. The balloon volume was adjusted with water to maintain an LV end-diastolic pressure (LVEDP) of 5 mmHg. A catheter was inserted into the pulmonary artery to collect coronary venous return for measuring coronary flow. The heart was paced at 222 beats/min during the ischemic period with an electronic stimulator (SEN-3201, Nihon Kohden, Tokyo, Japan).

### Experimental protocol

**Fig. 1:**
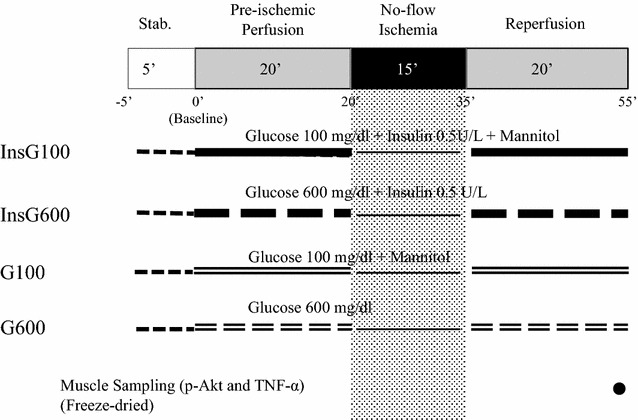
Experimental protocol. The InsG100 group received 0.5 U/L insulin, 100 mg/dL glucose and 27.5 mmol/L of mannitol in KH buffer. The InsG600 group received 0.5 U/L insulin and 600 mg/dL glucose in KH buffer. The G600 group was perfused with KH buffer containing 600 mg/dL

Following a stabilization period of 5 min, baseline hemodynamics were recorded. Firstly, the hearts were then randomly divided into three groups (n = 10) using a computer-generated random number table (MS Excel 2010): InsG100, InsG600, and G600 groups. The InsG100 group received 0.5 U/L insulin and 100 mg/dL glucose in KH buffer 20 min before no-flow ischemia (15 min) and during 20 min of reperfusion. The InsG600 group received 0.5 U/L insulin and 600 mg/dL glucose prior to no-flow ischemia and reperfusion. The G600 group received 600 mg/dL glucose throughout. To clarify the cardioprotective effect of insulin, we added G100 after examination of the three groups was completed. The G100 group received 100 mg/dL glucose throughout. To match the osmotic pressure of the InsG600 and G600 group, 27.5 mmol/L of mannitol was added to the solution in the InsG100 and G100 group. The experimental design is illustrated in Fig. 1.

### Measurements

Heart rate (bpm), maximum LV pressure derivative (LV dP/dt max) (mmHg/s) and rate-pressure product (RPP = LV systolic pressure × Heart Rate) were recorded continuously. Coronary flow (mL/min) was measured by timed collection of the perfusate (baseline, after 20 min of preconditioning, and after 1, 5, 10, 15, and 20 min of reperfusion) from the catheter inserted into the pulmonary artery.

Following perfusion and 20 min of reperfusion, the whole rat heart was quickly frozen in liquid nitrogen and freeze-dried for 6 days to measure p-Akt/total-Akt and TNF-α in the myocardial muscle.

The myocardium was suspended in assay lysis buffer (Lysis Buffer 6, R&D Systems, Minneapolis, MN, USA) containing phenylmethanesulfonyl fluoride (2 mM, Sigma-Aldrich, Inc., St. Louis, MO, USA) and protease inhibitor cocktail (Sigma-Aldrich, Inc.). The samples were then homogenized using a micro homogenizing system (MicroSmash MS-100R, TOMY SEIKO Co., LTD., Tokyo, Japan). The homogenates were centrifuged for 5 min at 2000*g*, and the supernatants were assayed for p-Akt by enzyme-linked immunosorbent assay (ELISA; The Invitrogen™ AKT [pS473] Elisa kit, Thermo Fisher Scientific, Camarillo, CA, USA) and total-Akt by ELISA; The Invitrogen™ AKT [total] Elisa kit, Thermo Fisher Scientific). The concentrations of p-Akt and total-Akt were quantified photometrically (Spectra Max 340, Molecular Devices, Sunnydale, CA, USA) at an absorbance of 450 nm. Wavelength correction was set to 540 nm. The values were expressed as Unit/μg of ratio p-Akt to total-Akt level.

To measure myocardial TNF-α content, the myocardium was suspended in normal saline. The samples were then homogenized using a micro homogenizing system (MicroSmash MS-100R, TOMY SEIKO Co., LTD.). The homogenates were centrifuged for 5 min at 2000*g*, and the supernatants were assayed by sandwich ELISA (Invitrogen rat TNF-a ELISA Kit, Life Technologies, Carlsbad, CA, USA) and quantified photometrically (Spectra Max 340, Molecular Devices) at an absorbance of 450 nm. The values were expressed as pg of TNF-α per gram of dry heart weight.

### Statistical analysis

The data are presented as the mean ± SD. Intragroup and intergroup comparisons in hemodynamics were analysed using two-way analysis of variance (ANOVA) followed by the Bonferroni post hoc test. Intergroup comparisons for baseline measurements and the concentrations of TNF-α and p-Akt/total-Akt were made with one-way ANOVA followed by the Bonferroni post hoc test. Two-sided *P* values <0.05 were considered statistically significant. All statistical analyses were performed using SPSS 21 for Windows (IBM, Chicago, IL).

## Results

**Fig. 2:**
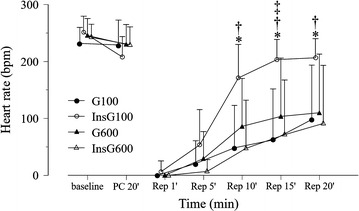
Changes in heart rate over time, before and after ischemia, in the four groups (n = 10). The data are presented as the mean ± SD. *HR* heart rate (bpm). *P < 0.05 vs. G100. ^†^ *P* < 0.05 vs. InsG600. ^‡^ *P* < 0.05 vs. G600

**Fig. 3:**
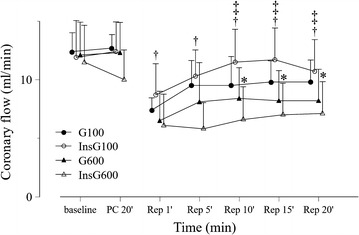
Changes in coronary flow over time, before and after ischemia, in the four groups (n = 10). The data are presented as the mean ± SD. *P < 0.05 vs. G100. ^†^ *P* < 0.05 vs. InsG600. ^‡^ *P* < 0.05 vs. G600

**Fig. 4:**
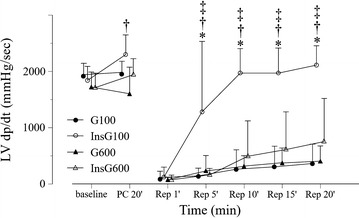
Changes in LV dP/dt max over time, before and after ischemia, in the four groups (n = 10). The data are presented as the mean ± SD. *P < 0.05 vs. G100. ^†^ *P* < 0.05 vs. InsG600. ^‡^ *P* < 0.05 vs. G600. *LV dP/dt max (mmHg/s)* maximum of left ventricular derivative of pressure development

**Fig. 5:**
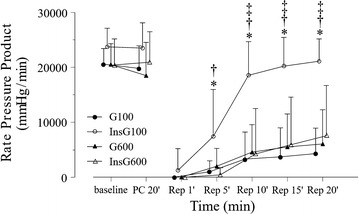
Changes in rate pressure product over time, before and after ischemia, in the four groups (n = 10). The data are presented as the mean ± SD. * *P* < 0.05 vs. G100. ^†^ *P* < 0.05 vs. InsG600. ^‡^ *P* < 0.05 vs. G600

There was no significant difference in baseline values among the groups (Table 1). Heart rate increased gradually after reperfusion in all groups (Fig. 2). In the InsG100 group, the heart rate was significantly higher than in the InsG600 group and G100 group at 10, 15, and 20 min after reperfusion and the G600 group after 15 min of reperfusion. Coronary flow changes are shown in Fig. 3. Coronary flow in the G600 group was significantly lower than in the G100 group after 10, 15, and 20 min of reperfusion. In the InsG100 group, coronary flow was significantly higher than in the InsG600 group at 1, 5, 10, 15, and 20 min after reperfusion and the G600 group at 10, 15, 20 min of reperfusion. In the InsG100 group, LV dP/dt max is significantly higher than after administration of insulin prior to ischemia compared with the G600 group (Fig. 4). After reperfusion, LV dP/dt max in the InsG100 group was significantly elevated when compared with the G100, InsG600 and G600 group at 5, 10, 15, and 20 min (Fig. 4). The rate-pressure product in the InsG100 group was significantly higher than in the other three groups (Fig. 5). There was no difference among the groups in the cardiac muscle TNF-α concentration (Table 2). The myocardial p-Akt/total-akt in the InsG100 group was significantly higher than in the other three groups after 20 min of reperfusion. The p-Akt/total-Akt in the InsG600 group were significantly higher than in the G100 group and the G600 group (Table 2).

**Table 1:** Baseline measurements

	G100	InsG100	G600	InsG600
Number (n)	10	10	10	10
Dry heart weight (g)	0.21 ± 0.04	0.21 ± 0.03	0.24 ± 0.03	0.22 ± 0.03
Heart rate (bpm)	232 ± 27	251 ± 29	250 ± 30	242 ± 23
dP/dt max (mmHg/s)	1940 ± 201	1920 ± 246	1720 ± 266	1710 ± 256
Coronary flow (mL/min)	12.4 ± 1.6	11.9 ± 1.3	12.1 ± 2.8	11.5 ± 3.1

Data are the mean ± SD

There are no significant differences among the groups

Baseline measurements are presented in absolute values as obtained 5 min after stabilization, except for dry heart weight, which was measured at the end of the experiment

*LV dP/dt max* maximum of left ventricular derivative of pressure development

**Table 2:** Myocardial TNF-α and p-Akt contents at the end of reperfusion

	G100	InsG100	G600	InsG600
Number (n)	10	10	10	10
Myocardial TNF-α contents (pg/g dry heart weight)	1568 ± 458	2073 ± 424	1925 ± 229	1787 ± 464
Myocardial p-Akt/total Akt (Unit/mg)	11.4 ± 11.9	77.9 ± 19.6*^,†,‡^	15.9 ± 10.9	53.6 ± 20.7*^,‡^

Data are the mean ± SD

There was no difference among the groups in the cardiac muscle TNF-α concentration 20 min after reperfusion. The myocardial p-Akt/total-Akt in the InsG100 group was significantly higher than the other two groups and that in the InsG600 group was significantly higher we than those in the InsG100 and InsG600 at 20 min after reperfusion

* *P* < 0.05 vs. G100

^†^ *P* < 0.05 vs. InsG600

^‡^ *P* < 0.05 vs. G600

## Discussion

The result of the present study indicates that acute hyperglycemia blunts the cardioprotective effects of pre-ischemic insulin pre-conditioning most likely mediated by the impairment of Akt activation.

Myocardial pre-conditioning aimed at reversing or mitigating the adverse consequences of cardiopulmonary bypass or ischemia can be achieved by short and repetitive ischemic periods or by the use of specific pharmacological agents. Ischemic pre-conditioning has long been recognized as effective strategy against subsequent, prolonged, and lethal ischemic assaults [1, 8]. Pharmacological options include the pre-emptive administration of volatile anesthetics, adenosine, nicorandil, delta opioids and nitroglycerin [2]. Recently, insulin also demonstrated cardioprotective qualities when given prior to an ischemic insult [3, 9].

Animal data suggest that acute hyperglycemia, a typical metabolic response to major surgical tissue trauma, diminishes the benefits of ischemic pre-conditioning [10]. Furthermore, high blood glucose concentrations suppressed positive effects of pre-conditioning techniques using isoflurane [11] or remote ischemia [12]. In agreement with the results of the present study hyperglycemia blunted the benefits of insulin regarding infarct size, necrosis and apoptosis following myocardial ischemia/reperfusion [13]. In contrast to Yu’s protocol [13] we used a reversible stunned rat heart model to mimic the context of cardiopulmonary bypass.

Insulin is a well established activator of the Akt signalling cascade which serves as important mediator of ischemic pre-conditioning [1]. In a constitutively active Akt mutant rat model Akt activation dramatically improved myocardial function after transient ischemia [14].

After binding to the insulin receptor it activates tyrosine kinase resulting in the phosphorylation of a number of tyrosine insulin receptor substrates (IRS) which in turn upregulate downstream effectors of PI3K, particularly Akt. [14, 15] Cardiomyocytes perfused with insulin had elevated p-Akt levels prior to ischemia compared with those that did not receive insulin [3]. Similarly, in the present study, insulin pre-conditioning was associated with increased myocardial p-Akt concentrations. Consistent with previous observations [13, 16] hyperglycemia decreased Akt phosphorylation suggesting that this pathway plays a key role in the inhibitory effect of hyperglycemia on insulin-induced preservation of myocardial contractility after ischemia.

Conversely, Baranyai et al. showed that a longer period of hyperglycemia (35 vs. 20 min in the present protocol) before ischemia led to increased pAkt levels and the activation of the Akt-the mechanistic target of rapamycin (mTOR) pathway [12] suggesting that the duration of hyperglycemia may be also important.

The hyperglycemic response to surgery is the consequence of a stress induced over-production of so called counter-regulatory hormones (catecholamines, growth hormone, cortisol) and cytokines resulting in stimulated hepatic glucose production and tissue insulin resistance. Hyperglycemia itself further exacerbates inflammatory and oxidative responses, potentially initiating a vicious cycle [17, 18]. In contrast to the results of a previous study [3], tissue TNF-α, a known inhibitor of insulin-induced IRS-1 tyrosine phosphorylation and myocardial contractility [19], was not affected by insulin pre-conditioning. In our previous protocol, insulin had decreased the release of TNF-α into the coronary efferent fluid 5 min after reperfusion, while no effect was seen 15 min later [3]. In the current study myocardial TNF-α was measured 20 min after reperfusion only, supporting the previously made observation in a myocardial infarction heart model of two phases of reperfusion injury, an early one within the first 3 min and a later phase after 40–60 min [20].

We acknowledge some limitations of this study.

Coronary flow was different in the four groups and myocardial oxygen consumption was not measured. We, therefore, cannot exclude the possibility that altered blood flow was, at least in part, responsible for the reported changes.

Because we did not include a group of animals treated with a specific Akt inhibitor nor studied other pathways including the hexosamine or Akt-mTOR system [12, 13] the exact of role of Akt as a mediator of insulin-pre-conditioning and hyperglycemia remains to be determined.

## Conclusion

Possibly mediated through the suppression of myocardial Akt phosphorylation acute hyperglycemia inhibits the cardioprotective effects of insulin pre-conditioning in the isolated rat heart further emphasizing the importance of glycemic control in the context of insulin therapy and myocardial ischemia.

## Abbreviations

CPB: cardiopulmonary bypass
PI3K: phosphatidylinositol 3-kinase
Akt: protein kinase B
p-Akt: phospho-protein kinase B
TNF-α: tumor necrosis factor-α
KH: Krebs–Henseleit
LV: left ventricular
LVEDP: left ventricular end-diastolic pressure
InsG100: 0.5 U/L insulin and 100 mg/dL glucose
InsG600: 0.5 U/L insulin and 600 mg/dL glucose
G600: 600 mg/dL glucose
G100: 100 mg/dL glucose
LV dP/dt max: maximum left ventricular pressure derivative
RPP: rate pressure product
ELISA: enzyme-linked immunosorbent assay
ANOVA: analysis of variance
IRS: insulin receptor substrates
mTOR: mechanistic target of rapamycin

## References

[1] Hausenloy DJ, Yellon DM (2009). Preconditioning and postconditioning: underlying mechanisms and clinical application. Atherosclerosis.

[2] Najmaii S, Redford D, Larson DF (2006). Hyperglycemia as an effect of cardiopulmonary bypass: intra-operative glucose management. J Extra Corpor Technol.

[3] Sato T, Sato H, Oguchi T, Fukushima H, Carvalho G, Lattermann R (2014). Insulin preconditioning elevates p-Akt and cardiac contractility after reperfusion in the isolated ischemic rat heart. Biomed Res Int.

[4] Duncan AE, Abd-Elsayed A, Maheshwari A, Xu M, Soltesz E, Koch CG (2010). Role of intraoperative and postoperative blood glucose concentrations in predicting outcomes after cardiac surgery. Anesthesiology.

[5] LaDisa JF, Krolikowski JG, Pagel PS, Warltier DC, Kersten JR (2004). Cardioprotection by glucose-insulin-potassium: dependence on KATP channel opening and blood glucose concentration before ischemia. Am J Physiol Heart Circ Physiol.

[6] Esposito K, Nappo F, Marfella R, Giugliano G, Giugliano F, Ciotola M (2002). Inflammatory cytokine concentrations are acutely increased by hyperglycemia in humans: role of oxidative stress. Circulation.

[7] Van den Berghe G (2004). How does blood glucose control with insulin save lives in intensive care?. J Clin Invest.

[8] Murry CE, Jennings RB, Reimer KA (1986). Preconditioning with ischemia: a delay of lethal cell injury in ischemic myocardium. Circulation.

[9] Iliadis F, Kadoglou N, Didangelos T (2011). Insulin and the heart. Diabetes Res Clin Pract.

[10] Kersten JR, Schmeling TJ, Orth KG, Pagel PS, Warltier DC (1998). Acute hyperglycemia abolishes ischemic preconditioning in vivo. Am J Physiol.

[11] Kehl F, Krolikowski JG, Mraovic B, Pagel PS, Warltier DC, Kersten JR (2002). Hyperglycemia prevents isoflurane-induced preconditioning against myocardial infarction. Anesthesiology.

[12] Baranyai T, Nagy CT, Koncsos G, Onódi Z, Károlyi-Szabó M, Makkos A (2015). Acute hyperglycemia abolishes cardioprotection by remote ischemic perconditioning. Cardiovasc Diabetol.

[13] Yu Q, Zhou N, Nan Y, Zhang L, Li Y, Hao X (2014). Effective glycaemic control critically determines insulin cardioprotection against ischaemia/reperfusion injury in anaesthetized dogs. Cardiovasc Res.

[14] Matsui T, Tao J, del Monte F, Lee KH, Li L, Picard M (2001). Akt activation preserves cardiac function and prevents injury after transient cardiac ischemia in vivo. Circulation.

[15] Bertrand L, Horman S, Beauloye C, Vanoverschelde JL (2008). Insulin signaling in the heart. Cardiovasc Res.

[16] Su H, Sun X, Ma H, Zhang HF, Yu QJ, Huang C (2007). Acute hyperglycemia exacerbates myocardial ischemia/reperfusion injury and blunts cardioprotective effect of GIK. Am J Physiol Endocrinol Metab.

[17] Dungan KM, Braithwaite SS, Preiser JC (2009). Stress hyperglycaemia. Lancet.

[18] Van Cromphaut SJ (2009). Hyperglycaemia as part of the stress response: the underlying mechanisms. Best Pract Res Clin Anaesthesiol.

[19] Kleinbongard P, Schulz R, Heusch G (2011). TNFalpha in myocardial ischemia/reperfusion, remodeling and heart failure. Heart Fail Rev.

[20] Povlsen JA, Lofgren B, Dalgas C, Jespersen NR, Johnsen J, Botker HE (2014). Frequent biomarker analysis in the isolated perfused heart reveals two distinct phases of reperfusion injury. Int J Cardiol.

